# Array-CGH diagnosis in ovarian failure: identification of new molecular actors for ovarian physiology

**DOI:** 10.1186/s13048-016-0272-5

**Published:** 2016-10-03

**Authors:** Sylvie Jaillard, Linda Akloul, Marion Beaumont, Houda Hamdi-Roze, Christele Dubourg, Sylvie Odent, Solène Duros, Nathalie Dejucq-Rainsford, Marc-Antoine Belaud-Rotureau, Célia Ravel

**Affiliations:** 1 CHU Rennes, Service de Cytogénétique et Biologie Cellulaire, F-35033 Rennes, France; 2 INSERM U1085-IRSET, Université de Rennes 1, F-35042 Rennes, France; 3 CHU Rennes, Service de Génétique Clinique, CLAD Ouest, F-35033 Rennes, France; 4 CHU Rennes, Service de Génétique Moléculaire et Génomique, F-35033 Rennes, France; 5 UMR 6290-Institut de Génétique et Développement, Université de Rennes 1, F-35043 Rennes, France; 6 Département de Gynécologie Obstétrique et Reproduction Humaine, CHU Rennes, F-35033 Rennes, France; 7 CHU Rennes, Service de Biologie de la Reproduction CECOS, 16 Bd de Bulgarie, F-35033 Rennes, France

**Keywords:** Copy number variations, Ovarian failure, Genetics of infertility, Anti-müllerian hormone

## Abstract

**Background:**

Ovarian failure (OF) is considered premature if it occurs before the age of 40.

This study investigates the genetic aetiology underlying OF in women under the age of 40 years.

**Methods:**

We conducted an experimental prospective study performing all genome microarrays in 60 patients younger than 40 years presenting an OF revealed by a decrease of circulating Anti-Müllerian Hormone (AMH) and leading to an oocyte donation program.

**Results:**

We identified nine significant copy number variations (CNVs) including candidate genes potentially implicated in reproductive function. These genes are principally involved in cell division and chromosome segregation (*SYCE1, CLASP1, CENP-A, CDC16*), in ciliary development and/or function (*RSPH1, KIF24*), are linked with known gonadal genes or expressed in female genital tract (*CSMD1, SEMA6D, KIAA1324*).

**Conclusions:**

Our data strengthen the idea that microarrays should be used in combination with karyotype for aetiological assessment of patients with OF. This analysis may have a therapeutic impact as the identification of new molecular actors for gonadal development or ovarian physiology is useful for the prediction of an ovarian reserve decline and makes possible preventive fertility preservation.

## Background

OF is characterized by an absence or a diminution of the normal ovarian physiology and is considered premature if it occurs before the age of 40 [1]. Premature ovarian failure (POF) is the end stage of this OF characterized by progressive cessation of the ovarian function with temporary or intermittent amenorrhea associated with elevated Follicle-Stimulating Hormone (FSH) concentration or low AMH dosage. The combined FSH and AMH observations are of clinical relevance in infertile women [2]. Indeed, serum AMH dosage is able to detect a diminished ovarian pool occurring before the onset of FSH elevation and the ultimate deficiency leading to amenorrhea [3, 4]. Some authors tried to redefine each step of this OF, from diminished ovarian reserve to POF, both of states leading to a poor ovarian response in IVF stimulations [5].

OF affects 1 % of women, reaching 30 % in some familial cases. Its occurrence varies considerably with the ethnic origin (1 % in Caucasians, 1.4 % in Afro-Americans, 1.4 % in Hispanics, 0.5 % in Chinese, and 0.1 % in Japanese), highlighting genetic factors implication [6]. Pathogenesis of an ovarian deficiency may be an initial decrease in the primordial follicle pool or an anticipated depletion of the primordial follicular pool or an altered follicular maturation [7]. Among genetic causes, chromosomal abnormalities occur in 8.8 to 33 % of women with POF depending on the population and size of the study. Ten to fifteen percent of cases are X chromosome abnormalities, mainly Turner Syndrome or X abnormalities such as X deletions, X inversions, isochromosomes or X-autosome translocations. These abnormalities can be found on the karyotype. *FMR1* premutation is another frequent genetic etiology that has to be searched for in the initial assessment. Other genetic etiologies can be suggested by associated symptoms. Several genes (autosomal or X-linked) potentially involved in primordial follicles activation and in folliculogenesis have been identified: *NR5A1*, *FIGLA*, *NOBOX*, *FOXO3*, *FOXO1*, *GDF9*, *BMP15*, *FOXL2*, *NANOS3*, *STAG3* or genes involved in hormonal regulation. Other genes have only been suspected to have such function: *SOHLH1*, *SOHLH2*, *LHX8*, *KIT/KITL*, *PTEN*, *BCL2*, *BAX* or *AKT/PIK3C* pathway [7–10]. Identification of genomic imbalances can provide insights into the involvement of genes or chromosomal regions in the occurrence of OF. As conventional karyotype cannot detect genomic imbalances <5 Mb in size (microdeletions and microduplications), microarrays offer an alternative for the identification of that type of copy number variations (CNVs). We describe in this study the results of the genetic testing by array-CGH of 60 patients with OF younger than 40 years presenting an unusual ovarian deficiency phenotype (low ovarian reserve and poor ovarian response to conventional stimulation). The main hormonal selective criterion in our study is the level of AMH rather than basal FSH (Table 1). The genetic analyses were performed both for diagnosis purpose (identification of known pathogenic gene) and research purpose (identification of candidate genes for female infertility).

**Table 1:** Candidate regions for POF phenotype (region, type of CNV, genomic coordinates hg19, minimal size, candidate gene), phenotypic and hormonal features of the OF patients presenting with a significant CNV variant

Patient	Region	Type of CNV	Genomic coordinates (hg19)	CNV minimal size	Candidate gene	Clinical features besides OF	AMH (ng/ml)	FSH (UI/l)	Age (years)
7	8p23.2	dup	2,308,926–5,935,998	3627 kb	*CSMD1*	Spaniomenorrhea	0.9	6.9	21
						3 miscarriages			
20	2q14.2q14.3	dup	122,121,531–122,863,325	742 kb	*CLASP1*	Spaniomenorrhea	0.4	24	34
						1 ectopic pregnancy			
						Breast cancer in an aunt			
21	2p23.3	dup	26,557,453–27,116,447	559 kb	*CENP A*	Bronchiectasis	0.9	9.7	31
21	21q22.3	del	43,762,549–43,985,429	223 kb	*RSPH1*	Ciliar anomalies of the ducts			
						Failure of 3 ART cycles			
25	13q34	dup	114,931,625–115,043,128	112 kb	*CDC16*	Ovarian cyst	<0.4	8.6	34
						Failure of 6 ART cycles			
						Hypothyroidy			
29	9p13.3	dup	34,206,594–34,391,999	185 kb	*KIF24*	Secondary amenorrhea	<0.4	13	30
31	1p13.31	del	109,697,100–109,745,781	49 kb	*KIAA1324*	Secondary amenorrhea	0.14	133	34
43	10q26.31	dup	135,254,039–135,377,532	123 kb	*SYCE1*	Secondary amenorrhea	<0.4	81	28
47	15q21.1	dup	48,057,293–48,145,309	88 kb	*SEMA6D*	Vaginal and uterine septum	<0.4	na	35

*ART* assisted reproductive technics, *na* not available

## Methods

### Patients

Sixty patients with OF were included in this study. Women were recruited from Rennes Hospital and all participants gave their written informed consent for genetic investigations according to French law and DNA sample biobanking (CRB Germetheque). Patients with *FRM1* premutation or chromosomal aberrations were excluded unless the latter did not explain the phenotype (one polymorphic chromosome 2 pericentric inversion and one der(15;22) robertsonian translocation). The patients were infertile women under the age of 40 years, presenting a poor ovarian response in in vitro fertilization (IVF) treatment combined with the decrease of AMH dosage (<1 ng/ml) and all enrolled in an oocyte donation program. There were 4 familial cases of infertility or OF. The average age of OF patients was 30-year-old, primary amenorrhea occurred in four cases, secondary amenorrhea in 11 cases and there was no amenorrhea in 45 cases (spaniomenorrhea or irregular periods). Material of the patients’ parents was not available. Anyway, inherited CNVs would probably have been discarded if there was no familial history of infertility because causative variants are under strong negative selection. Nevertheless, it is not possible to completely exclude those inherited CNVs, particularly from the parent with the opposite sex chromosome, as gonad development in males and females involves distinct genes and pathways [11].

### Karyotype

Conventional R-banded karyotypes were performed on metaphase cells prepared from PHA-stimulated cultured peripheral blood cells according to standard protocols [12].

### Array-CGH

Oligonucleotide array-CGH was performed using the Agilent Human Genome CGH microarray 180 K (Agilent Technologies, Santa Clara, CA, USA). These microarrays are constituted of 180 000 60-mer oligonucleotide probes that span both coding and non-coding regions. Coverage of the human genome is made with a median probe spacing of 13 kb. The experiments were performed according to version 7.1 (December 2011) of the protocol provided by Agilent (Agilent Oligonucleotide Array-Based CGH for Genomic DNA Analysis). Reference genomic DNA (gDNA) was the female one provided by Agilent. Patients’ gDNAs were obtained from total blood of the patients using the Janus Varispan Automated Workstation (Perkin Elmer, Waltham, MA, USA) and the Macherey Nagel technology Nucleospin Blood L (Macherey-Nagel, Düren, Germany). gDNAs concentrations were measured with a Nanodrop ND-1000 spectrophotometer (Nanodrop Technologies, Wilmington, DE, USA). The Agilent SurePrint G3 CGH Bundle was used for restriction digestion, random priming labeling to differentially label test and reference gDNAs with Cy5-dUTP and Cy3-dUTP respectively, hybridization (Human Cot-1 DNA, Blocking Agent and Hybridization Buffer). After denaturation and pre-annealing at 37 °C for 30 min, the hybridization mixture was deposited on the microarray slide and hybridized in a hybridization chamber for 24 h in a 67 °C rotating oven. Then washing steps were performed. Microarrays were scanned using the Agilent scanner G2565BA. Images were analysed using Agilent Feature Extraction Software version 10.7.3.1 (CGH_107_Sep09 protocol) permitting creation of QC reports for each patient where the value of the Derivative Log Ratio Spread (DLRS), which is the spread of the derivative log ratio values, was used as a quality criterion. A graphical overview and analysis of the data were obtained by using the Agilent CytoGenomics software version 3.0.6.6. Identification of probes with a significant gain or loss was based on the log2 ratio plot deviation from 0 with cut-off values of 0.5 to 1 and -0.5 to -1 respectively. Aberrant signals including five or more adjacent probes were considered as genomic aberrations and were retained for further studies, including FISH, if they were not or infrequently listed as polymorphisms in the Database of Genomic Variants or DGV (http://dgv.tcag.ca/dgv/app/home) used as a control population. This DGV lists common CNVs detected in more than 10 000 healthy individuals. Nevertheless, information from this database have to be interpreted with caution since it represents healthy individuals without information concerning fertility. The CNVs are described using the version GRCh37 of the human genome (hg19).

### FISH analysis

FISH was performed using BAC clones to confirm the CNVs observed in array-CGH. Clones were selected on the public database UCSC Genome Browser. The selected clones were obtained from the BACPAC Resource Center (Children’s Hospital Oakland Research Institute, Oakland, CA, USA). Bacterial culture was performed in LB medium supplemented with chloramphenicol (12.5 μg/mL) and DNA from the clones was isolated using a Nucleobond PC 20 kit (Macherey Nagel, Düren, Germany). BAC DNA was labeled with SpectrumGreen-dUTP (Abbott Molecular Inc., Downers Grove, IL, USA) or with Cyanine3-dCTP (Amersham Biosciences, Buckinghamshire, UK) by random priming using the Bioprime Array CGH Genomic Labeling System (Invitrogen, Carlsbad, CA, USA). FISH was performed according to standard procedures [13]. After hybridization, the slides were counterstained with 4,6-diamino-2-phenyl-indol or DAPI (Qbiogene MP Biomedicals, Santa Ana, CA, USA). Metaphase slides were analysed with an epifluorescent microscope Olympus BX61 (Olympus, Rungis, France) equipped with a charge-coupled device camera. Images were captured using Isis® software (MetaSystems, Altlussheim, Germany).

### AMH level screening

To avoid any discrepancy by using different assay methods, all measurements of serum AMH level were performed using the automated Access AMH assay (Beckman-Coulter, Brea, CA, USA) in the same laboratory.

## Results

A total of 263 CNVs (146 deletions and 117 duplications) ranging in size from 20 kb to 3.6 Mb were identified in the 60 gDNA samples (average: 4.4 per patient). The CNVs identified in the study were compared with CNVs reported in DGV. 244 CNVs were excluded because they have been described as frequent variants in DGV. Finally, 19 CNVs were considered significant (7.2 % of the CNVs, detection rate in the patients: 32 %) and observed in 13 patients: eight deletions and 11 duplications. Ten CNVs (3.8 % of the CNVs, detection rate in the patients: 16.6 %) corresponded to Variants Of Uncertain Significance (VOUS, variants for which associations with clinical features may be weak or mixed in previous reports or deletions of gene involved in recessive disorders) and nine CNVs (3.4 % of the CNVs, detection rate in the patients: 15 %) were candidate. They were further described in the discussion, irrespective of the type of CNV even if genomic losses are more likely to result in a clinical phenotype than genomic gains (Table 1).

## Discussion

We have used whole-genome array-CGH to identify microdeletions and microduplications in a cohort of 60 women presenting an OF. Search for genomic imbalances by microarray in patients with OF has become challenging and allowed in previous studies the identification of CNVs throughout the genome, on X chromosome and other regions. These rearrangements could affect known gonadal genes or candidate gonadal genes [11, 14–17]. As indicated in the study of McGuire et al., these CNVs are frequently located on autosomes suggesting that candidate genes for OF are located on them [14]. Aboura et al. have shown, in their study of 99 patients, 8 statistically different CNVs from control population, in 1p21.1, 5p14.3 (*DNAH5*), 5q13.2 (*NAIP*), 6p25.3 (*DUSP22*), 14q32.33 (*AKT1*), 16p11.2 (*NUPR1*), 17q12, and Xq28, with five genes potentially involved in reproduction [15]. Ledig et al. identified 44 significant CNVs in 74 POF and ovarian dysgenesis, with genes involved in meiosis as *PLCB1, RB1CC1, MAP4K4*, genes involved in DNA repair as *RBBP8* and genes involved in folliculogenesis or male fertility as *IMMP2L, FER1L6, MEIG1* [16]. McGuire et al. identified 24 autosomal CNVs in 89 patients, with genes linked to abnormal reproductive phenotypes in mouse models (*SYCE1* in 10q26.3 and *CPEB1* in 15q25.2) [14]. Norling et al. have shown 11 significant CNVs in 26 POF, most of them involving new regions and candidate genes (e.g. *MCTP2* on 15q26.2, *CACNAIC* on 12p13.33) [17]. In the present study in 60 women presenting OF, nine candidate CNVs were likely to be involved in fertility. Anomalies of genes potentially involved in primordial follicles activation or folliculogenesis might explain the occurrence of this feature.

### CNVs already linked to POF

A 3.6 Mb 8p23.2 duplication involving the *CSMD1* gene (*Cub and sushi multiple domains 1,* OMIM 608397) was identified in patient 7. A 680 kb duplication involving this gene was observed in the study of McGuire et al. (patient POF-45) [14]. Expression analyses have shown that CSMD1 can be expressed in follicles and corpus luteum of the female reproductive system [18]. These data (recurrence of the variant and follicular expression) suggest that *CSMD1* could play a role in folliculogenesis. However, *CSMD1* has also been associated with autistic spectrum disorders and schizophrenia and is a known target of mir-137, a microRNA that regulates neuronal maturation and adult neurogenesis [19]. Futhermore, *CSMD1*, as tumor suppressor gene, has been associated with head and neck squamous cell carcinoma and with the tumorigenesis of several other epithelial cancers, including breast cancer with rare variants (deletions) predisposing individuals to breast cancer [20].

In the study of McGuire et al. [14], a 160 kb deletion at 10q26.3 (including *CYP2E1* and *SYCE1*) was potentially linked to the POF phenotype. In our study, a 123 kb duplication of the same region was observed in patient 43 suggesting a role of this variant in gonadal development. *SYCE1* (*Synaptonemal complex central element 1*, OMIM 611486) has been previously shown in animal models to be linked with the occurrence of POF. It is also involved in chromosome synapsis and recombination during meiosis. Recently, a homozygous mutation in *SYCE1* was identified by exome sequencing in two sisters with POF [21].

### CNVs involving other genes associated to cell division and chromosome segregation

Several genes encode proteins involved in cohesion, kinetochores and motor functions. It is supposed that CNVs of these genes could lead to errors in meiosis and gametogenesis. In patient 20, a 741 kb duplication at 2q14 involving the 5′end of *CLASP1* (*CLIP-associated protein 1*, OMIM 605852) was detected. CLASP proteins are non-motile proteins associated with microtubules and interacting with CLIP proteins. *CLASP1* is required for the kinetochore-microtubule interaction and allows the mitotic spindle to exhibit normal dynamic behavior. It remains at the kinetochores upon the completion of chromosome congression and throughout anaphase, suggesting that it may modulate the dynamics of attached microtubules throughout mitosis [22]. CLASP1-Astrin-Kif2b complex acts as a central switch at kinetochores that defines mitotic progression and promotes fidelity by temporally regulating kinetochore-microtubule attachments [23]. Based on studies in the Xenopus meiotic spindle, Xorbit/CLASP also appears as a critical factor linking chromosome segregation to microtubules dynamics during meiosis [24].

In patient 25, a 113 kb duplication at 13q34 involving *CDC16* (*Cell division cycle 16*, OMIM 603461) was observed. *CDC16* is one of several subunits of the Anaphase-Promoting Complex (APC), which functions at the metaphase-to-anaphase transition of the cell cycle and is regulated by spindle checkpoint proteins. It is a core tetratricopeptide repeat (TPR)-containing protein stabilized by CDC26 in the APC and it is supposed that the stable complex CDC26-CDC16 may serve as a platform for assembling higher-order multi-TPR complex required for APC function [25]. It has been showed that CDC16 is essential for cell division in human cells with a knockdown leading to a mitotic arrest [26]. Defect in APC/C activity seems to be involved in the waves of oocyte degeneration occurring during shift from mitosis to meiosis, final stages of meiotic prophase I and at the breakdown of the oocyte nests preluding the primordial follicle assembly [27]. Furthermore, CDC16 is part of TBC1D7 (Tre2-Bub2-Cdc16-1 domain family member 7), a subunit of the Tuberous Sclerosis Complex (TSC) in the PI3K/AKT/mTORC1 pathway [28] for which some of the key components have implicated in spermatogenesis and oogenesis [29].

In patient 21, a 559 kb duplication at 2p23.3 involving *CENP-A* (*Centromere protein A*, OMIM 117139) was observed. CENP-A is a histone H3-like protein involved in centromeric nucleosome formation. In mouse, Cenpa is essential for kinetochore targeting of Cenpc and plays an early role in organizing centromeric chromatin at interphase. The chromosomes seen in the null *Cenpa* embryos appeared morphologically more condensed and scattered than those of normal embryos and no discernible mitotic chromosomes were apparent in the 6.5-day null embryos examined, suggesting cessation of mitosis at this point. The study suggests a critical epigenetic function for *CENP-A* in marking a chromosomal region for centromere formation [30].

### CNVs involving genes associated with ciliary development and/or function

In patient 21, additionally to the *CENP-A* duplication, a 21q22.3 223 kb deletion involving *RSPH1* (*Radial spoke head 1 homolog*, OMIM 609314) was identified. *RSPH1* is involved in primary ciliar dyskinesia (PCD), a disorder with male infertility due to dysmotility of spermatozoa and reduced fertility or a history of ectopic pregnancies in affected women. PCD is a genetically heterogeneous, autosomal-recessive disorder with mutations reported in 28 genes (most frequently *DNAH5*). Loss-of-function mutations of *RSPH1* have been associated to a milder clinical phenotype, as compared with patients with PCD with typical ultrastructural defects or mutations in genes that are commonly associated with PCD [31]. It is interesting to note that patient 21 presents bronchiectasis and ciliary anomalies of the ducts which can be explained by loss-of-function of this gene and lead us to sequence the second allele of this gene.

In patient 29, a 185 kb duplication at 9p13.3 involving *UBAP1* (OMIM 609787), *KIF24* (OMIM 613747), *NUDT2* (OMIM 602852), *KIAA 1161* and *C9orf24* was observed. Among these genes, *KIF24* (*Kinesin family member 24*, OMIM 613747) encodes a kinesin-13 motor protein that preferentially localizes to the distal end of mother centrioles. It possesses microtubule-depolymerizing activity and regulates cilia formation in cycling cells by depolymerizing centriole microtubules. In quiescent cells, *KIF24* is detected at the basal body of the primary cilium and its overexpression suppresses ciliogenesis [32]. Thus *KIF24* has a role in the regulation of ciliogenesis which is important during embryonic development and in adult physiology [33].

### CNVs involving genes linked with known gonadal genes or with expression in female genital tract

In patient 47, a 88 kb duplication at 15q21.1 was identified. This CNV involve the 5′ end of *SEMA6D* (*Semaphorin 6D*, OMIM 609295), a gene potentially linked with *FOXO1*. It has indeed been showed that two potential targets of *SEMA6D*, the transcription factors *FOS* and *FOXO1*, were both increased in SEMA6D-high patients in case of breast cancer [34]. A gain or loss of function of S*EMA6D* could then result in a dysregulation in folliculogenesis as *FOXO1*, expressed in human luteinized mural granulosa cells, is supposed to be involved in human folliculogenesis and luteinization [35].

A 44 kb deletion at 1p13.31 involving *KIAA1324* (*Estrogen-induced gene 121 or EIG121*, OMIM 611298) was identified in patient 31. *KIAA1324* has a moderate expression in ovary; nevertheless, it is known to be induced by estrogen in the female reproductive tract [36].

## Conclusions

The next step to support the hypothesis of the causative role of a candidate gene would be a large-scale screening of individuals with OF, to identify similar CNVs or point mutations. More patients have to be studied to assess the clinical implication of the genes which implies several further studies to obtain a sufficient cohort. Futhermore, functional analyses would be necessary to confirm the involvement of the candidate genes in the OF before they should become valuable markers of OF and then used in diagnosis.

The identification of CNVs in OF by array-CGH has become an indispensable tool for clinical setting, facilitating patient management and genetic counseling. Here we have shown that investigations of dosage imbalances for known genes should be recommended in young patients presenting an incipient OF detectable on low levels of AMH whatever the level of FSH is. However, array-CGH can detect submicroscopic CNVs across the whole genome but it cannot detect balanced chromosomal rearrangements. A combined approach, including karyotype and array-CGH is then required to deliver an accurate genetic diagnosis in a clinical setting [37]. Next generation sequencing technologies are now emerging and will provide new insights into the genetics of gonadal pathologies [10, 21, 38, 39]. Increased understanding of the molecular basis will arise from a greater understanding of the genes that may be implicated in the development of ovarian deficiency and identifying the molecular pathways that may be defective. These data will help to diagnose the failure earlier, and therefore provide a way to save or protect remaining functional follicles before the development of OF. The recent introduction of oocyte vitrification has significantly advanced the outcome of oocyte cryopreservation, leading to clinical results comparable to those achieved in IVF using fresh oocytes. Consequently, we will be able to propose the preservation of the reproductive potential for these individuals or others members of their family, making genetic counseling more effective.

## Abbreviations

AMH: Anti-müllerian hormone
CGH: Comparative genomic hybridization
CNVs: Copy number variations
DGV: Database of genomic variants
FISH: Fluorescent in situ hybridization
FSH: Follicle-stimulating hormone
IVF: In vitro fertilization
OF: Ovarian failure
PCD: Primary ciliar dyskinesia
POF: Premature ovarian failure
TPR: Tetratricopeptide repeat
VOUS: Variants of uncertain significance

## References

[1] Ravel C, Kazdar N, Leveque J (2016). Ovarian failure: new treatments in perspective?. Gynecol Obstet Fertil.

[2] Gleicher N, Kim A, Kushnir V, Weghofer A, Shohat-Tal A, Lazzaroni E, Lee HJ, Barad DH (2013). Clinical relevance of combined FSH and AMH observations in infertile women. J Clin Endocrinol Metab.

[3] Van Disseldorp J, Faddy MJ, Themmen AP, de Jong FH, Peeters PH, van der Schouw YT, Broekmans FJ (2008). Relationship of serum antimüllerian hormone concentration to age at menopause. J Clin Endocrinol Metab.

[4] Broer SL, Eijkemans MJ, Scheffer GJ, van Rooij IA, de Vet A, Themmen AP, Laven JS, de Jong FH, Te Velde ER, Fauser BC, Broekmans FJ (2011). Anti-mullerian hormone predicts menopause: a long-term follow-up study in normoovulatory women. J Clin Endocrinol Metab.

[5] Cohen J, Chabbert-Buffet N, Darai E (2015). Diminished ovarian reserve, premature ovarian failure, poor ovarian responder--a plea for universal definitions. J Assist Reprod Genet.

[6] Bashamboo A, Ravel C, Brauner R, McElreavey K (2009). NR5A1 and ovarian failure. Med Sci (Paris).

[7] Wood MA, Rajkovic A (2013). Genomic markers of ovarian reserve. Semin Reprod Med.

[8] Ledig S, Hiort O, Scherer G, Hoffmann M, Wolff G, Morlot S, Kuechler A, Wieacker P (2010). Array-CGH analysis in patients with syndromic and non-syndromic XY gonadal dysgenesis: evaluation of array CGH as diagnostic tool and search for new candidate loci. Hum Reprod.

[9] Baronchelli S, Villa N, Redaelli S, Lissoni S, Saccheri F, Panzeri E, Conconi D, Bentivegna A, Crosti F, Sala E, Bertola F, Marozzi A, Pedicini A, Ventruto M, Police MA, Dalprà L (2012). Investigating the role of X chromosome breakpoints in premature ovarian failure. Mol Cytogenet.

[10] Fonseca DJ, Patiño LC, Suárez YC, de Jesús Rodríguez A, Mateus HE, Jiménez KM, Ortega-Recalde O, Díaz-Yamal I, Laissue P (2015). Next generation sequencing in women affected by nonsyndromic premature ovarian failure displays new potential causative genes and mutations. Fertil Steril.

[11] White S, Ohnesorg T, Notini A, Roeszler K, Hewitt J, Daggag H, Smith C, Turbitt E, Gustin S, van den Bergen J, Miles D, Western P, Arboleda V, Schumacher V, Gordon L, Bell K, Bengtsson H, Speed T, Hutson J, Warne G, Harley V, Koopman P, Vilain E, Sinclair A (2011). Copy number variation in patients with disorders of sex development due to 46,XY gonadal dysgenesis. PLoS One.

[12] Dutrillaux B, et Couturier J (1981). La pratique de l’analyse chromosomique.

[13] Muleris M, Richard F, Apiou F, Dutrillaux B (1996). Hybridation in situ en cytogénétique moléculaire.

[14] McGuire MM, Bowden W, Engel NJ, Ahn HW, Kovanci E, Rajkovic A (2011). Genomic analysis using high-resolution single-nucleotide polymorphism arrays reveals novel microdeletions associated with premature ovarian failure. Fertil Steril.

[15] Aboura A, Dupas C, Tachdjian G, Portnoï MF, Bourcigaux N, Dewailly D, Frydman R, Fauser B, Ronci-Chaix N, Donadille B, Bouchard P, Christin-Maitre S (2009). Array comparative genomic hybridization profiling analysis reveals deoxyribonucleic acid copy number variations associated with premature ovarian failure. J Clin Endocrinol Metab.

[16] Ledig S, Röpke A, Wieacker P (2010). Copy number variants in premature ovarian failure and ovarian dysgenesis. Sex Dev.

[17] Norling A, Hirschberg AL, Rodriguez-Wallberg KA, Iwarsson E, Wedell A, Barbaro M (2014). Identification of a duplication within the GDF9 gene and novel candidate genes for primary ovarian insufficiency (POI) by a customized high-resolution array comparative genomic hybridization platform. Hum Reprod.

[18] Kraus DM, Elliott GS, Chute H, Horan T, Pfenninger KH, Sanford SD, Foster S, Scully S, Welcher AA, Holers VM (2006). CSMD1 is a novel multiple domain complement-regulatory protein highly expressed in the central nervous system and epithelial tissues. J Immunol.

[19] Cukier HN, Dueker ND, Slifer SH, Lee JM, Whitehead PL, Lalanne E, Leyva N, Konidari I, Gentry RC, Hulme WF, Booven DV, Mayo V, Hofmann NK, Schmidt MA, Martin ER, Haines JL, Cuccaro ML, Gilbert JR, Pericak-Vance MA (2014). Exome sequencing of extended families with autism reveals genes shared across neurodevelopmental and neuropsychiatric disorders. Mol Autism.

[20] Kuusisto KM, Akinrinade O, Vihinen M, Kankuri-Tammilehto M, Laasanen SL, Schleutker J (2013). Copy number variation analysis in familial BRCA1/2-negative Finnish breast and ovarian cancer. PLoS One.

[21] de Vries L, Behar DM, Smirin-Yosef P, Lagovsky I, Tzur S, Basel-Vanagaite L (2014). Exome sequencing reveals SYCE1 mutation associated with autosomal recessive primary ovarian insufficiency. J Clin Endocrinol Metab.

[22] Maiato H, Fairley EA, Rieder CL, Swedlow JR, Sunkel CE, Earnshaw WC (2003). Human CLASP1 is an outer kinetochore component that regulates spindle microtubule dynamics. Cell.

[23] Manning AL, Bakhoum SF, Maffini S, Correia-Melo C, Maiato H, Compton DA (2010). CLASP1, astrin and Kif2b form a molecular switch that regulates kinetochore-microtubule dynamics to promote mitotic progression and fidelity. EMBO J.

[24] Hannak E, Heald R (2006). Xorbit/CLASP links dynamic microtubules to chromosomes in the Xenopus meiotic spindle. J Cell Biol.

[25] Wang J, Dye BT, Rajashankar KR, Kurinov I, Schulman BA (2009). Insights into anaphase promoting complex TPR subdomain assembly from a CDC26-APC6 structure. Nat Struct Mol Biol.

[26] Kittler R, Putz G, Pelletier L, Poser I, Heninger AK, Drechsel D, Fischer S, Konstantinova I, Habermann B, Grabner H, Yaspo ML, Himmelbauer H, Korn B, Neugebauer K, Pisabarro MT, Buchholz F (2004). An endoribonuclease-prepared siRNA screen in human cells identifies genes essential for cell division. Nature.

[27] Klinger FG, Rossi V, De Felici M (2015). Multifaceted programmed cell death in the mammalian fetal ovary. Int J Dev Biol.

[28] Dibble CC, Cantley LC (2015). Regulation of mTORC1 by PI3K signaling. Trends Cell Biol.

[29] Baker MD, Ezzati M, Aloisio GM, Tarnawa ED, Cuevas I, Nakada Y, Castrillon DH (2014). The small GTPase Rheb is required for spermatogenesis but not oogenesis. Reproduction.

[30] Howman EV, Fowler KJ, Newson AJ, Redward S, MacDonald AC, Kalitsis P, Choo KH (2000). Early disruption of centromeric chromatin organization in centromere protein A (Cenpa) null mice. Proc Natl Acad Sci U S A.

[31] Knowles MR, Ostrowski LE, Leigh MW, Sears PR, Davis SD, Wolf WE, Hazucha MJ, Carson JL, Olivier KN, Sagel SD, Rosenfeld M, Ferkol TW, Dell SD, Milla CE, Randell SH, Yin W, Sannuti A, Metjian HM, Noone PG, Noone PJ, Olson CA, Patrone MV, Dang H, Lee HS, Hurd TW, Gee HY, Otto EA, Halbritter J, Kohl S, Kircher M, Krischer J, Bamshad MJ, Nickerson DA, Hildebrandt F, Shendure J, Zariwala MA (2014). Mutations in RSPH1 cause primary ciliary dyskinesia with a unique clinical and ciliary phenotype. Am J Respir Crit Care Med.

[32] Kobayashi T, Tsang WY, Li J, Lane W, Dynlacht BD (2011). Centriolar kinesin Kif24 interacts with CP110 to remodel microtubules and regulate ciliogenesis. Cell.

[33] Ware SM, Aygun MG, Hildebrandt F (2011). Spectrum of clinical diseases caused by disorders of primary cilia. Proc Am Thorac Soc.

[34] Chen D, Li Y, Wang L, Jiao K (2015). SEMA6D expression and patient survival in breast invasive carcinoma. Int J Breast Cancer.

[35] Pisarska MD, Kuo FT, Tang D, Zarrini P, Khan S, Ketefian A (2009). Expression of forkhead transcription factors in human granulosa cells. Fertil Steril.

[36] Schlumbrecht MP, Xie SS, Shipley GL, Urbauer DL, Broaddus RR (2011). Molecular clustering based on ERα and EIG121 predicts survival in high-grade serous carcinoma of the ovary/peritoneum. Mod Pathol.

[37] Jaillard S, Bashamboo A, Pasquier L, Belaud-Rotureau MA, McElreavey K, Odent S, Ravel C (2015). Gene dosage effects in 46, XY DSD: usefulness of CGH technologies for diagnosis. J Assist Reprod Genet.

[38] Eggers S, Smith KR, Bahlo M, Looijenga LH, Drop SL, Juniarto ZA, Harley VR, Koopman P, Faradz SM, Sinclair AH (2015). Whole exome sequencing combined with linkage analysis identifies a novel 3 bp deletion in NR5A1. Eur J Hum Genet.

[39] Laissue P (2015). Aetiological coding sequence variants in non-syndromic premature ovarian failure: From genetic linkage analysis to next generation sequencing. Mol Cell Endocrinol.

